# High-Intensity Exercise Training Alters the Effect of *N*-Acetylcysteine on Exercise-Related Muscle Ionic Shifts in Men

**DOI:** 10.3390/antiox12010053

**Published:** 2022-12-27

**Authors:** Anders K. Lemminger, Matteo Fiorenza, Kasper Eibye, Jens Bangsbo, Morten Hostrup

**Affiliations:** The August Krogh Section for Human Physiology, Department of Nutrition, Exercise and Sports, University of Copenhagen, 1165 Copenhagen, Denmark

**Keywords:** ROS, oxygen species, scavengers, NAC, high-intensity training, potassium, lactate, pH, antioxidant, performance

## Abstract

This study investigated whether high-intensity exercise training alters the effect of *N*-acetylcysteine (a precursor of antioxidant glutathione) on exercise-related muscle ionic shifts. We assigned 20 recreationally-active men to 6 weeks of high-intensity exercise training, comprising three weekly sessions of 4–10 × 20-s all-out bouts interspersed by 2 min recovery (SET, n = 10), or habitual lifestyle maintenance (n = 10). Before and after SET, we measured ionic shifts across the working muscle, using leg arteriovenous balance technique, during one-legged knee-extensor exercise to exhaustion with and without *N*-acetylcysteine infusion. Furthermore, we sampled vastus lateralis muscle biopsies for analyses of metabolites, mitochondrial respiratory function, and proteins regulating ion transport and antioxidant defense. SET lowered exercise-related H^+^, K^+^, lactate^−^, and Na^+^ shifts and enhanced exercise performance by ≈45%. While *N*-acetylcysteine did not affect exercise-related ionic shifts before SET, it lowered H^+^, HCO_3_^−^, and Na^+^ shifts after SET. SET enhanced muscle mitochondrial respiratory capacity and augmented the abundance of Na^+^/K^+^-ATPase subunits (α_1_ and β_1_), ATP-sensitive K^+^ channel subunit (Kir6.2), and monocarboxylate transporter-1, as well as superoxide dismutase-2 and glutathione peroxidase-1. Collectively, these findings demonstrate that high-intensity exercise training not only induces multiple adaptations that enhance the ability to counter exercise-related ionic shifts but also potentiates the effect of *N*-acetylcysteine on ionic shifts during exercise.

## 1. Introduction

Intense exercise causes major ionic shifts in skeletal muscle that contribute to muscle fatigue [1,2,3,4,5]. Exercise training enhances the ability to counter exercise-related ionic shifts (e.g., lactate^−^, H^+^, K^+^, and Na^+^) and improves the performance of the trained muscle [6,7,8,9,10,11]. This is particularly true for high-intensity interval-based training forms performed at intensities above those corresponding to maximal oxygen uptake (V̇O_2max_), such as speed endurance training (SET) [3,5]. But, while several studies have investigated the effect of high-intensity training forms on muscle ionic shifts, our understanding of the multitude of systems involved is less clear. During exercise, muscle-derived reactive oxygen species (ROS) induce modifications of proteins modulating ion transport systems and energy turnover, ultimately accelerating muscle fatigue [12,13]. Accordingly, the favorable adaptations elicited by SET may not only stem from an upregulation of ion transport proteins but from training-induced enhancements in intrinsic ROS-scavenging capacity of the muscle [14,15].

Infusion of the antioxidant *N*-acetylcysteine can alleviate the detrimental effects of ROS via its scavenging action. Studies in well-trained individuals show that *N*-acetylcysteine attenuates exercise-induced decline in Na^+^/K^+^-ATPase activity and preserves K^+^ balance, thereby leading to enhanced performance [16,17]. However, this effect may be training-dependent. Change in performance with *N*-acetylcysteine correlated (*r* = 0.78) with V̇O_2max_ in a small sample size of seven subjects with a clear separation of untrained from trained subjects [18], and *N*-acetylcysteine has been shown to increase the rise in plasma K^+^ concentration-to-work ratio during intense intermittent exercise in untrained men, which is indicative of impaired K^+^ regulation [19]. These findings imply that training potentiates the effect of *N*-acetylcysteine on exercise-related muscle ionic shifts but this warrants further investigation in a prospective training study with recreationally active individuals.

Given that metabolic perturbations exacerbate ionic shifts [3,4,5], adaptations that pertain to oxidative capacity likely contribute to the improved regulation of ionic shifts elicited by a period of high-intensity training. Specifically, a higher mitochondrial respiratory capacity would lower the reliance on anaerobic energy pathways and preserve muscle metabolic stability during exercise. Because SET enhances muscle mitochondrial respiratory capacity [20,21], it may well be that mitochondrial functional adaptations contribute to the greater ability to counter exercise-related ionic shifts associated with a period of SET.

In this study, we investigated putative mechanisms underlying SET-induced enhancement of muscle ion handling during exercise, including adaptations in ion transport proteins, antioxidant enzymes, and mitochondrial respiratory capacity. We also examined whether SET altered the effect of *N*-acetylcysteine on exercise-related ionic shifts across the working muscles. We hypothesised that SET not only would improve the ability to counter exercise-related ionic shifts but also potentiate the effect of *N*-acetylcysteine on ionic shifts during exercise.

## 2. Methods and Materials

### 2.1. Subjects and Ethics

Twenty recreationally active men completed this study. Inclusion criteria were healthy males, 18–40 yrs old, maximal oxygen consumption (V̇O_2max_) between 45–55 mL·min^−1^·kg^−1^, and BMI between 19–26 kg·m^−2^. Exclusion criteria were abnormal electrocardiogram, chronic disease, ongoing medical treatment, allergic reaction to study drugs, and smoking. Females were not included due to potential confounding effects of menstrual cycle-dependent fluctuations in sex hormones which may affect proteins regulating muscle ionic shifts and ultimately exercise training-related outcomes [22]. Before inclusion, subjects received written and oral information about the content of the study and potential risks and discomforts associated with the experimental procedures. Subjects were allocated to either an intervention group undergoing a SET period (SET, n = 10) or a control group maintaining a habitual lifestyle (CON, n = 10). Subject characteristics are presented in Table 1. The study was approved by the National Committee on Health Research Ethics (H-17004045) and conducted in accordance with the Declaration of Helsinki (2013). The study was registered at clinicaltrials.gov (NCT03317704).

### 2.2. Study Design

This study was conducted as a single-blinded longitudinal randomised controlled trial and was part of a larger study investigating the physiological adaptations to high-intensity exercise training [20].

### 2.3. Preliminary Procedures

Before inclusion, subjects underwent a screening visit to assure the eligibility criteria were met. After receiving the informed consent, V̇O_2max_ was measured by indirect calorimetry during an incremental test on a bike ergometer (Monark LC4, Monark Exercise, Vansbro, Sweden) as previously described [20]. After a short break, subjects were familiarised with the one-legged knee-extensor exercise model. The familiarisation protocol consisted of 3 × 2-min bouts at 12, 18, and 24 W, respectively, and was performed with both legs. Subjects were instructed to actively contract the quadriceps during knee extension while relaxing the thigh during knee flexion and maintaining a cadence of 60 RPM.

Two to four days after the screening, subjects returned to the laboratory for determination of leg peak power output (W_max_). W_max_ was determined during an incremental one-legged knee-extensor exercise test to exhaustion. The test started at 12 W and workload was increased by 6 W every min until exhaustion, which was defined as an inability to maintain a cadence of 60 RPM for 10 s or a drop in cadence below 55 RPM. Thereafter, subjects were allocated to either CON or SET and underwent two experimental days, which were repeated after the intervention. In SET, W_max_ was (mean ± SD) 73.9 ± 12.8 W in the right leg (SET_Saline_) and 72.6 ± 13.3 W in the left leg (SET_NAC_). In CON, W_max_ was 70.3 ± 17.4 W in the right leg (CON_Saline_) and 67.0 ± 15.5 W in the left leg (CON_NAC_). Subjects recorded their food intake 48 h before experimental day 1 and replicated the same food intake before each experimental day. In addition, subjects refrained from caffeine, alcohol, and exercise 24 h before the experimental days.

### 2.4. Experimental Setup

**Figure 1:**
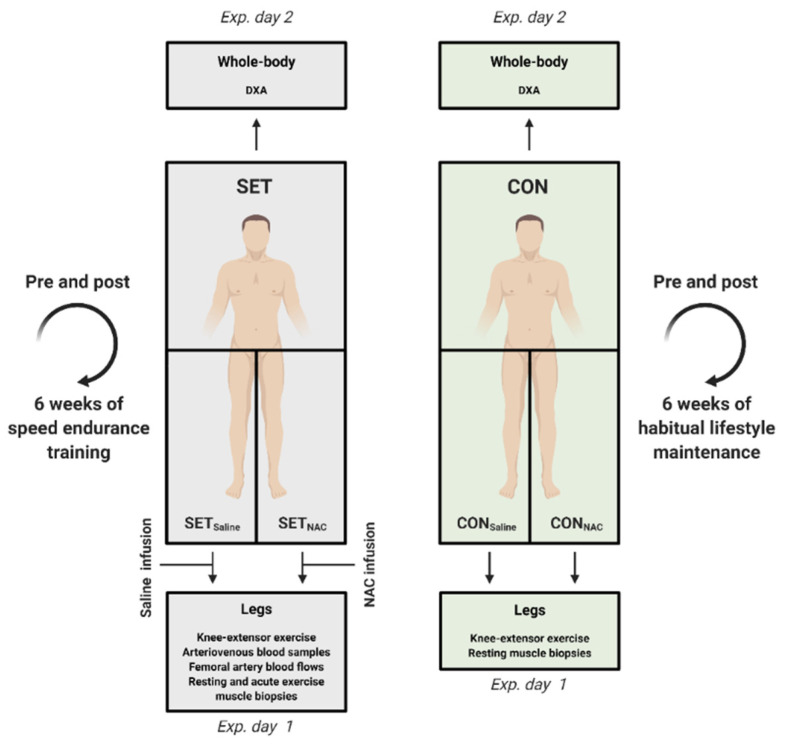
Schematic overview of the experimental protocol. Exp. day, experimental day; DXA, dual-energy X-ray absorptiometry; NAC, *N*-acetylcysteine; SET, speed endurance training group; SET_Saline_, right leg in SET (i.e., leg receiving saline infusion before and after the SET intervention); SET_NAC,_ left leg in SET (i.e., leg receiving NAC infusion before and after the SET intervention); CON, control group; CON_Saline_, right leg in CON; CON_NAC_, left leg in CON.

A schematic overview of the experimental design is presented in Figure 1.

#### 2.4.1. Experimental Day 1

Subjects reported to the laboratory in the morning, after an overnight fast, and ingested a standardised meal (204 Kcal; 18.7 g carbohydrates, 6.8 g fats, 17.0 g protein) with ad libitum water and rested in a supine position for 15 min. Then, a 4 mm incision was made in the vastus lateralis muscle under local anaesthesia (Xylocaine 20 mg·mL^−1^ without epinephrine, AstraZeneca, London, UK) and a muscle biopsy was collected using a Bergström needle with suction. Afterward, subjects in the SET group had catheters (20 gauge, Teleflex, Wayne, PA, USA) inserted into the femoral vein of both legs and femoral artery in one of the legs under local anaesthesia (Xylocaine 20 mg·mL^−1^ without epinephrine). Catheters were inserted below the inguinal ligament and advanced in the proximal direction using ultrasound-guided Doppler (Vivid E9, GE, Healthcare, Waukesha, WI, USA) to ensure correct placement. After ~60 min of rest in the supine position, subjects were seated on a Krogh ergometer with a hip angle of 110° and rested for an additional 10 min period. Thereafter, saline was infused at a constant rate (1 mL·h^−1^) in the left femoral vein and subjects performed the one-legged knee-extensor exercise protocol with the right leg (SET_Saline_). The protocol included three bouts of submaximal exercise consisting of 5 min at 30% W_max_, 5 min at 50% W_max_, and 4 min at 80% W_max_. Upon completion of the last submaximal exercise bout, subjects carried out a high-intensity exercise bout consisting of increments of 6 W·min^−1^ until exhaustion, which was determined as described above. Then, subjects rested for 2 h in a supine position to ensure minimal carry-over effects from the previous exercise of the contralateral leg [23]. After the resting period, a resting muscle biopsy was taken from the left vastus lateralis muscle as described above. *N*-acetylcysteine infusion was initiated in the right femoral vein at 125 mg·kg^−1^·h^−1^ for 15 min to reach peak plasma *N*-acetylcysteine concentrations [17]. The infusion rate was then reduced to 25 mg·kg^−1^·h^−1^, as previously described [17]. This intravenous administration protocol has been shown to achieve a plasma concentration of NAC sufficient to produce a pharmacological effect while avoiding adverse effects [24]. Following an additional 5 min at a low *N*-acetylcysteine infusion rate, the left leg (SET_NAC_) performed the same one-legged knee-extensor exercise protocol as the right leg (SET_Saline_). Immediately after exercise termination, a muscle biopsy was taken from the m. vastus lateralis of the exercising leg. Blood was drawn from the femoral artery and vein at rest and during the last minute of each submaximal bout. After the high-intensity bout, blood was drawn every minute until exhaustion. In addition, blood was drawn immediately after exhaustion and 0.5, 1, 2, and 4 min into recovery. Venous blood was sampled ~5 s after the arterial blood to account for the mean transit time of the arterial blood through the capillary bed [25]. The femoral arterial blood flow was measured at the blood-sampling time-points using ultrasound Doppler. Due to hardware malfunction, data from the SET_NAC_ of one subject were excluded.

#### 2.4.2. Experimental Day 2

Two to four days after experimental day 1, subjects reported to the laboratory after an overnight fast and had their body composition measured by dual-energy X-ray absorptiometry (DXA) scans (Lunar iDXA, GE Healthcare, GE Medical systems, Belgium, Bruxelles). Before scanning, subjects rested in a supine position for 10 min to allow fluid distribution, thereby reducing intra-scan variation [26,27]. In addition, two DXA scans were performed to minimise scan-to-scan variation [28].

### 2.5. Training Intervention

Subjects underwent a six-week intervention period, as previously described [20]. Briefly, during the intervention period subjects were instructed to maintain their physical activity level and not to change their diet. In addition, subjects in SET performed three weekly training sessions of high-intensity interval training, specifically SET, on indoor spinning bikes. The SET protocol consisted of 20 s sprints interspersed with 2 min of active recovery, with the number of sprints progressively increasing from 4 to 10 during weeks 1–5, whereas a tapering period was adopted in week 6. Training sessions comprised a 7 min warm-up at ~70% HR_max_. All training sessions were supervised, and subjects wore chest strap heart rate monitors. Heart rate values were displayed by a projector and recorded (Polar Team2, Polar, Bethpage, NY, USA). The mean heart rate during training sessions was 81 ± 1% of HR_max_ and peaked at 91 ± 1% of HR_max_. Aside from one subject who missed one training session, training compliance was 100%.

### 2.6. Measurements and Data Analysis

#### 2.6.1. Body Composition

The composition of the thighs and whole body was calculated using software (enCORE Forma v. 15, GE Healthcare Lunar, Buckinghamshire, UK). The thigh was defined as the area from the ischial tuberosity to the patellar groove.

#### 2.6.2. Blood Samples and Blood Flow

Arterial and venous blood samples were drawn in heparinised syringes and were immediately analysed on a blood gas analyser (ABL800 FLEX, Radiometer, Copenhagen, DK) for plasma [lactate^−^], [K^+^], [Na^+^], [HCO_3_^−^], and pH as well as haemoglobin and haematocrit. At rest and during knee-extensor exercise at 30% and 50% of W_max_, duplicate samples were drawn and the mean of the two samples was used for data analysis.

Femoral arterial blood flow was measured using Doppler ultrasonography (Vivid E9) with a linear probe operating at an image frequency of 8.0 MHz and a Doppler frequency of 3.1 MHz as previously described [29]. Blood flow was measured over a 15 s period before and after each blood sampling with the average of these blood flows being used for data analysis. At exhaustion, the blood flow was only measured after blood sampling.

#### 2.6.3. Calculations

The net plasma ion exchange of the leg was calculated in accordance with previous studies [11,30] accounting for net transcapillary water exchange (*J_v_*) into or out of the vein:

$$Exchange_{ion}=\left(F_{p}-J_{v}\right)\cdot \;ion_{v}-F_{p}\cdot \;ion_{a}$$

where *ion_v_* and *ion_a_* are the venous and arterial ion concentrations, respectively.

Femoral arterial plasma flow (*F_p_*) was calculated as:

$$F_{p}=F\cdot \left(1-\frac{Hct_{a}}{100}\right)$$

where *F* is the femoral arterial blood flow and *Hct_a_* is the arterial haematocrit.

*J_v_* was calculated as:

$$J_{v}=F\cdot \left(\left[\left(\frac{Hb_{a}}{Hb_{v}}\right)\cdot \left(\frac{100-Hct_{v}}{100-Hct_{a}}\right)\right]-1\right)$$

where *Hb_a_* and *Hb_v_* are the arterial and venous haemoglobin concentration, respectively, and *Hct_v_* is the venous haematocrit.

To calculate H^+^ exchange as described above, arterial and venous H^+^ concentrations (*cH^+^*) were calculated based on pH and corrected for buffering of H^+^ by haemoglobin and HCO_3_^−^ in accordance with previous studies [6,31]:

$$cH^{+}=\left[1-\left(Hb\cdot 0.023\right)\right]\cdot \left(HCO_{3}{}^{-}-24.4\right)+\left(2.3\cdot Hb+7.7\right)\cdot \left(pH-7.4\right)$$

The arterial and venous *cH^+^* were calculated with the corresponding arterial or venous values of the given blood sample.

Total ionic shifts were calculated as the area under the curve (AUC) assuming that there was a linear relationship between adjacent measurements. AUC was calculated for ionic exchange during submaximal exercise (30, 50, and 80% W_max_) and the incremental part until exhaustion as well as the total ionic exchange during exercise.

#### 2.6.4. Muscle Biopsies

All muscle biopsies were collected through individual incisions separated by 1–2 cm. Muscle samples were snap-frozen in liquid nitrogen and stored at −80 °C. Before snap-freezing the resting muscle sample from the left leg, a small piece (~20 mg) was placed in ice-cold preservation solution (BIOPS) for determination of mitochondrial respiratory capacity, as previously described [32]. Frozen muscle samples were freeze-dried and dissected free from blood, connective tissue, and fat under a microscope. The dissected muscle was stored in tubes at −80 °C for subsequent analyses of protein content, lactate, pH, and buffer capacity.

#### 2.6.5. High-Resolution Respirometry

Within two hours of muscle biopsy sampling, the muscle tissue in BIOPS was prepared for high-resolution respirometry (Oxygraph-2k, Oroboros Instruments, Innsbruck, Austria), as previously described [32]. In brief, muscle tissue was dissected free from connective tissue and fat after which fibres were permeabilised in BIOPS containing saponin and subsequently washed twice in mitochondrial respiration medium (MiR06). High-resolution respirometry measurements were determined in duplicates at 37 °C using 1–3 mg wet weight muscle fibres per chamber in MiR06. The oxygen concentration was kept between 200 and 450 µM. Reoxygenation was done by injection of hydrogen peroxide into the chamber. To assess different mitochondrial respiratory states, a substrate-uncoupler-inhibitor titration (SUIT) protocol was used as previously described [33]. The following substrates were added: malate (2 mM) and octanoylcarnitine (0.2 mM) to support electron entry through fatty acid β-oxidation and complex I (CI) inducing leak respiration (L) in the absence of adenylates. Sub-saturating ADP (2.5 mM) was added to determine fatty acid oxidation (FAO). Submaximal CI-linked OXPHOS (CI_D_) was measured with the addition of glutamate (10 mM). Succinate (10 mM) was added to stimulate complex II (CII) thereby inducing submaximal CI+CII-linked OXPHOS (P_D_). Another titration of ADP (2.5 mM) was added to reach saturating levels for the determination of maximal CI+CII-linked OXPHOS (P). The addition of cytochrome C (10 µM) was made to test the mitochondrial outer membrane integrity which changed oxygen flux <10%. Carbonyl cyanide p-trifluoro-methoxyphenyl hydrazine (FCCP) (1.5–2.5 µM) was titrated in stepwise concentrations (0.5 µM) to reach the respiratory electron transfer-pathway capacity (E). CII-linked E-capacity (E_CII_) was achieved by the addition of rotenone (0.5 µM). Lastly, malonic acid (5 mM), myxothaizol (0.5 µM), and antimycin A (2.5 µM) were added to measure residual oxygen consumption (ROX). All respiratory data were corrected for ROX.

#### 2.6.6. Immunoblotting

Muscle protein content was determined by SDS-PAGE and Western blot, as previously described [20]. First, muscle samples were freeze-dried (~2 mg dry weight) and homogenised in a fresh batch of ice-cold buffer [10% glycerol, 20 mM Na-pyrophosphate, 150 mM NaCl, 50 mM HEPES (pH 7.5), 1% NP-40, 20 mM β-glycerophosphate, 2 mM sodium orthovanadate, 10 mM NaF, 2 mM PMSF, 1 mM EDTA (pH 8), 1 mM EGTA (pH 8), 10 µg·mL^−1^ aprotinin, 10 µg·mL^−1^ leupeptin, and 3 mM benzamidine]. Thereafter, samples were rotated for 1 h at 4 °C followed by centrifugation at 17,500× *g* for 20 min at 4 °C. The supernatant (lysate) was collected and a bovine serum albumin (BSA) standard kit (Thermo Fisher Scientific, Waltham, MA, USA) assayed in triplicate was used to determine total protein concentration in each sample. Then, lysate samples were diluted in double-distilled H_2_O and 6 × Laemmli buffer (7 mL 0.5 M Tris-base, 3 mL glycerol, 0.93 g DTT, 1 g SDS, and 1.2 mg bromophenol blue) to reach equal protein concentrations. Equal amounts of protein (range: 6–12 µg) were loaded in each well of 4–15% precast gels (Bio-Rad, Hercules, CA, USA). Samples from the same leg of the same subject were loaded on the same gel, with the samples from pre and post being loaded in adjacent wells. To allow gel-to-gel comparisons, the same pool of a mixed human muscle standard lysate was loaded in three different wells per gel and the mean intensity of these samples was used for normalisation, as previously described [34]. Proteins were separated by SDS-PAGE gel electrophoresis and semidry transferred to a PVDF membrane (MilliporeSigma, Burlington, MA, USA). The membranes were blocked in either 2–5% skim milk or 3% BSA in a mixture of Tris-buffered saline and Tween 20 (TBST) before overnight incubation at 4 °C in primary antibody diluted in either 2–5% skim milk or 3% BSA (Supplementary Table S1). After washing in TBST, membranes were incubated with a secondary horseradish peroxidase-conjugated antibody for ~1 h at room temperature. The secondary antibodies used were diluted 1:5000 in 2–5% skim milk or 3% BSA depending on the primary antibody (P-0447, P-0448, and P-0449; Agilent Technologies, Santa Clara, CA, USA). The membrane staining was visualised by incubation with a chemiluminescent horseradish peroxidase substrate (MilliporeSigma) before image digitalisation on a Chemi Doc MP (Bio-Rad). Western blot-band intensity was determined by densitometry quantification (total band intensity adjusted for background intensity) using Image Lab v.4.0 (Bio-Rad). Protein content was determined in duplicates (i.e., two different samples were obtained from the same muscle specimen after dissection and the mean value of the two samples was used).

#### 2.6.7. Muscle Lactate^−^

The muscle lactate^−^ concentration ([lactate^−^]) was determined in dry weight (d.w.) muscle tissue (~2.0 mg). Determination was made by extraction in 1.5 M perchloric acid, neutralisation to pH 7.0 with 2.2 M KHCO_3_, followed by fluorometric analyses of the supernatant as previously described (Kalsen et al., 2016). A reagent solution was created, which consisted of 1.5 M glycylglycine, 1.5 mM NAD^+^, and 10.2 mM glutamic acid, and stock solution of 4.0 mM lactate^−^ (Radiometer, Copenhagen, Denmark) was used to create a standard curve. The microplate reading was set at excitation at 355 nm with emission set at 460 nm (Fluoroskan Ascent, ThermoFischer Scientific, Waltham, MA, USA) which was read before and 60 min after glutamic-pyruvic transaminase (10105589001; Sigma-Aldrich, St. Louis, MO, USA) and LDH (10127876001; Sigma-Aldrich) was added to the samples.

#### 2.6.8. Muscle pH and Buffer Capacity

Muscle pH and buffer capacity were measured by a small glass electrode (N5800BNCS, Schott instruments) after homogenisation of ~1 mg freeze-dried sample in a nonbuffered solution containing 145 mM KCl, 10 mM NaCl, and 5 mM sodium fluoride [35]. Samples were adjusted to a pH of 7.1 with 0.01 M NaOH followed by a series of titrations with 0.01 M HCl until pH was <6.5, with the pH being measured after each titration. The non-HCO_3_^−^ physiochemical buffer capacity was calculated from moles of H^+^ required to change pH from 7.1 to 6.5 and is expressed as mmol H^+^·kg d.w.^−1^·pH^−1^ [35].

#### 2.6.9. Muscle Lactate^−^ and H^+^ Gradients

The intracellular muscle lactate^−^ and H^+^ was calculated as described previously [36]:

$$\left[C_{i}\right]=\frac{C_{m}-\left[C_{e}\right]\cdot 0.15}{\left(H_{2}O_{tot}-0.15\right)}$$

where *C_i_*, *C_m_*, and *C_e_* are the intracellular, muscle, and venous concentration, respectively. *H_2_O_tot_* is the water fraction in the biopsy and was assumed to be 78% [37] and 0.15 is the assumed factional interstitial volume of the total muscle water [36]. The intracellular-extracellular gradient was calculated as the difference between intracellular and extracellular concentration, with the venous plasma concentration being used as the best estimate for the extracellular concentration.

### 2.7. Statistical Analysis

Statistical analyses were performed in SPSS version 26 (IBM Corporation, Armonk, NY, USA). Data were deemed normally distributed based on Shapiro-Wilk’s test and Q-Q plots. A linear mixed model was used to estimate within and between-group changes (pre-post) with leg and time as fixed effects, whereas changes during submaximal exercise (30, 50, and 80% of W_max_) and 0.5–4 min into the recovery from exercise were assessed with leg, time, and sample as fixed effects. Thirty-two blood flow measurements of 401 (7.9%) and forty-four blood samples of 1060 (3.9%) were missing. Missing-at-random data were imputated using the mean of five multiple imputations. A standardised multivariate mixed model was used to assess changes (pre-post) with more than one series of longitudinal data. Correlations between main outcomes were assessed using Pearson’s correlation coefficient analysis. Absolute pre-post values are presented as mean ± SD. Repeated measurements are presented as mean ± SEM. Outcome statistics are presented as mean change with 95% confidence intervals (CI).

## 3. Results

### 3.1. Exercise Performance

**Figure 2:**
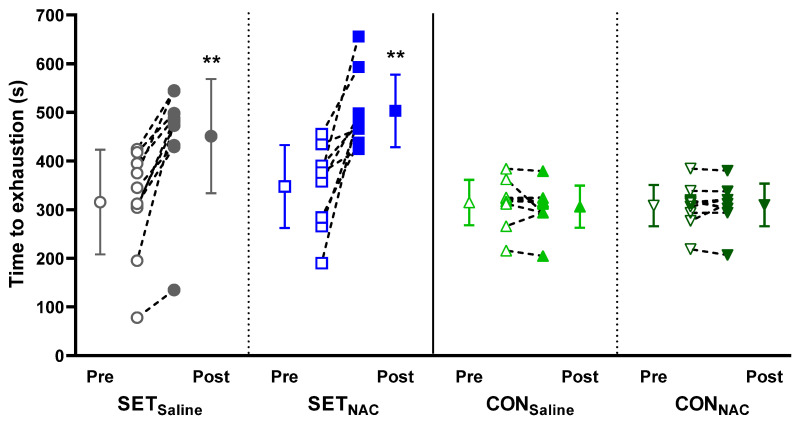
Leg exercise performance without and with *N*-acetylcysteine infusion before and after speed endurance training (SET). Time to exhaustion during knee-extensor exercise without (SET_Saline_, n = 10) and with *N*-acetylcysteine (SET_NAC_, n = 9) infusion before (Pre) and after (Post) SET as well as before and after habitual lifestyle maintenance (CON) for the right (CON_Saline_, n = 10) and left (CON_NAC_, n = 10) leg. ** Post different from Pre (*p* < 0.01). Data are presented as mean ± SD with individual changes.

SET increased time to exhaustion during incremental knee-extensor exercises to a similar extent in both experimental legs (Figure 2). For the right leg, where the knee-extensor exercise was performed during infusion of saline before and after the training period (SET_Saline_), performance was enhanced by 43% (16 to 70%; *p* < 0.001). For the left leg, where the exercise was performed during infusion of *N*-acetylcysteine before and after the training period (SET_NAC_), performance was enhanced by 45% (28 to 61%; *p* = 0.001). Performance did not change in CON.

### 3.2. Body Composition

SET increased thigh lean mass by 182 g (90 to 274 g; *p* = 0.001) and 185 g (82 to 289 g; *p* = 0.003) in SET_Saline_ and SET_NAC_, respectively, and also lowered body fat percentage (*p* < 0.05) (Table 2). No changes were observed in CON.

### 3.3. Femoral Plasma Flow

**Figure 3:**
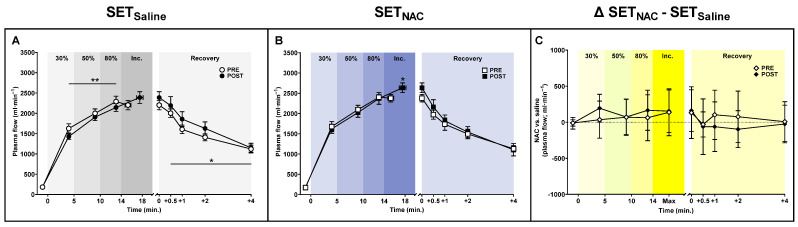
Effect of speed endurance training (SET) on femoral arterial plasma flow without and with *N*-acetylcysteine infusion. Femoral arterial plasma flow before, during, and after knee-extensor exercise without ((**A**), SET_Saline_, n = 10), and with *N*-acetylcysteine ((**B**), SET_NAC_, n = 9) infusion as well as difference (Δ) between SET_NAC_ and SET_Saline_ ((**C**), n = 9) before (Pre) and after (Post) SET. ** Post different from Pre (*p* < 0.05). * Post different from Pre (*p* < 0.05). Data are presented as mean ± SEM (SET_Saline_ and SET_NAC_) or mean ± 95% CI (Δ SET_NAC_ − SET_Saline_).

Since SET and *N*-acetylcysteine infusion may affect exercise hyperaemia and peripheral vascular function [20,38], changes in muscle blood flow must be accounted for when assessing exercise-related muscle ionic shifts. During submaximal exercise, mean femoral arterial plasma flow was 146 mL·min^−1^ (−244 to −48; *p* = 0.005) lower after the training period in SET_Saline_, whereas no training-induced changes were observed in SET_NAC_ (Figure 3). At exhaustion, femoral arterial plasma flow was non-significantly higher (−29 to 402 mL·min^−1^; *p* = 0.083) and higher (29 to 483 mL·min^−1^; *p* = 0.031) after the training period than before in SET_Saline_ and SET_NAC_, respectively. In recovery, mean femoral arterial plasma flow was higher after the training intervention in SET_Saline_ (*p* = 0.043), but not in SET_NAC_ (*p* = 0.383).

### 3.4. Plasma Lactate^−^ Shifts

**Figure 4:**
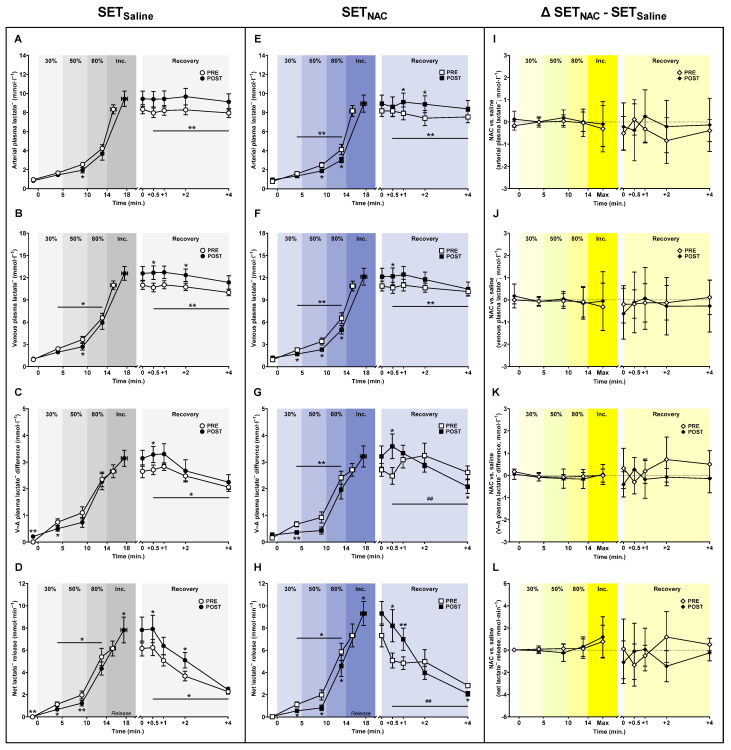
Effect of speed endurance training (SET) on plasma lactate^−^ shifts without and with *N*-acetylcysteine infusion. Plasma lactate^−^ concentrations (**A**–**C**,**E**–**G**) and net leg lactate^−^ exchange (**D**,**H**) before, during, and after knee-extensor exercise without (SET_Saline_, n = 10) and with *N*-acetylcysteine (SET_NAC_, n = 9) infusion as well as difference (Δ) between SET_NAC_ and SET_Saline_ (**I**–**L**, n = 9) before (pre) and after (post) SET. ** Post different from pre (*p* < 0.01). * Post different from pre (*p* < 0.05). ^##^ Trial × time effect (*p* < 0.01). Data are presented as mean ± SEM (SET_Saline_ and SET_NAC_) or mean ± 95% CI (Δ SET_NAC_ − SET_Saline_).

SET lowered arterial and venous plasma [lactate^−^] during submaximal exercise. Specifically, after the training intervention, arterial plasma [lactate^−^] was 0.6 mmol·L^−1^ (−1.2 to 0.0; *p* = 0.049) lower at 50% W_max_ in SET_Saline_, while it was 0.6 mmol·L^−1^ (−1.1 to −0.1; *p* = 0.023) and 1.1 mmol·L^−1^ (−2.1 to −0.1; *p* = 0.034) lower at 50 and 80% W_max_, respectively, in SET_NAC_ (Figure 4). Venous plasma [lactate^−^] was 0.5 mmol·L^−1^ (−1.0 to 0.1; *p* = 0.081) and 1.0 mmol·L^−1^ (−1.9 to −0.1; *p* = 0.040) lower at 30 and 50% W_max_, respectively, after the training intervention in SET_Saline_, while it was 0.5 (−1.0 to −0.1; *p* = 0.021), 1.1 (−2.0 to −0.2; *p* = 0.022), and 1.5 mmol·L^−1^ (−2.9 to −0.2; *p* = 0.029) lower at 30, 50, and 80% W_max_, respectively, after the training intervention in SET_NAC_. While arterial and venous plasma [lactate^−^] at exhaustion did not change with SET, lactate^−^ levels were greater in recovery after the training intervention in both SET_Saline_ and SET_NAC_ (*p* < 0.001).

The venous-arterial plasma [lactate^−^] difference was 0.2 (−0.5 to 0.0; *p* = 0.065), 0.4 (−0.7 to 0.0; *p* = 0.041), and 0.1 mmol·L^−1^ (−0.7 to 0.5; *p* = 0.763) lower at 30, 50, and 80% W_max_, respectively, after training in SET_Saline_, with corresponding venous-arterial differences being 0.3 (−0.5 to −0.1; *p* = 0.008), 0.5 (−1.0 to 0.1; *p* = 0.079), and 0.4 mmol·L^−1^ (−0.9 to 0.0; *p* = 0.063) lower at 30, 50, and 80% W_max_, respectively, after training in SET_NAC_. At exhaustion, the venous-arterial plasma [lactate^−^] difference was not significantly affected by training in SET_Saline_ (*p* = 0.109) and SET_NAC_ (*p* = 0.052). In recovery, the venous-arterial plasma [lactate^−^] difference was higher after the training intervention in SET_Saline_, but only in the immediate recovery (+0.5 min) for SET_NAC_.

The net leg lactate^−^ release during submaximal exercise was 0.5 (0.9 to 0.0; *p* = 0.048), 0.7 (1.2 to 0.3; *p* = 0.004), and 1.0 mmol·min^−1^ (2.2 to −0.1; *p* = 0.078) lower at 30, 50, and 80% W_max_, respectively, after the training intervention in SET_Saline_, with corresponding leg lactate^−^ release values being 0.6 (1.0 to 0.1; *p* = 0.018), 1.2 (2.3 to 0.0; *p* = 0.048), and 1.3 mmol·min^−1^ (2.3 to 0.2; *p* = 0.025) lower at 30, 50, and 80% W_max_, respectively, after training in SET_NAC_. At exhaustion, net leg lactate^−^ release was 1.7 mmol·min^−1^ (0.2 to 3.2; *p* = 0.034) and 2.0 mmol·min^−1^ (0.1 to 3.9; *p* = 0.041) higher after the training intervention in SET_Saline_ and SET_NAC_, respectively. In recovery, mean net leg lactate^−^ release was greater after the training intervention in SET_Saline_ and SET_NAC_, but only within the first minute for SET_NAC_.

### 3.5. Plasma pH Shifts

**Figure 5:**
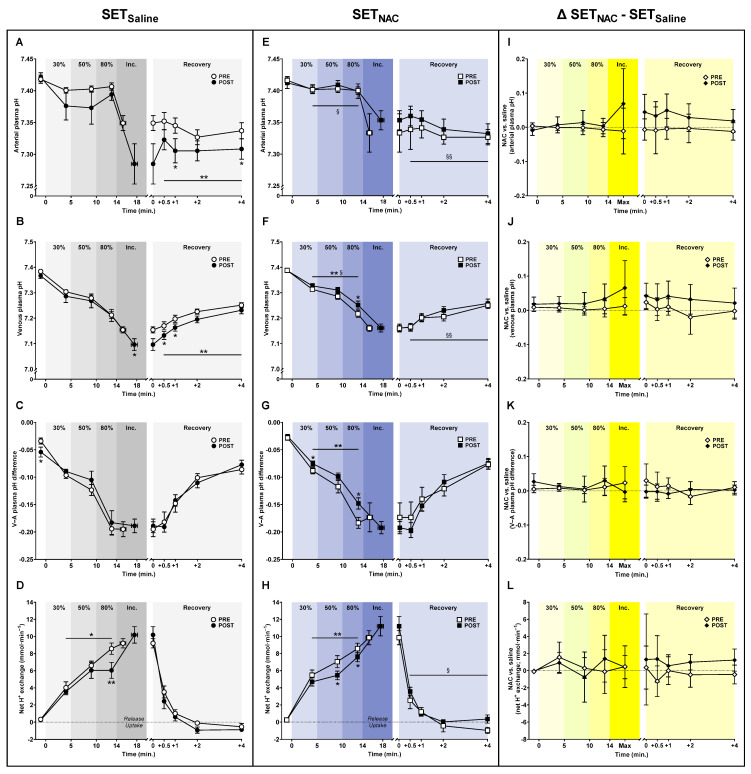
Effect of speed endurance training (SET) on plasma pH and H^+^ shifts without and with *N*-acetylcysteine infusion. Plasma pH (**A**–**C**,**E**–**G**) and net leg H^+^ exchange (**D**,**H**) before, during, and after knee-extensor exercise without (SET_Saline_, n = 10) and with *N*-acetylcysteine (SET_NAC_, n = 9) infusion as well as differences (Δ) between SET_NAC_ and SET_Saline_ (**I**–**L**, n = 9) before (pre) and after (post) SET. ** Post different from pre (*p* < 0.01). * Post different from pre (*p* < 0.05). ^§§^ Trial × leg effect (*p* < 0.01). ^§^ Trial × leg effect (*p* < 0.05). Data are presented as mean ± SEM (SET_Saline_ and SET_NAC_) or mean ± 95% CI (Δ SET_NAC_ − SET_Saline_).

The exercise-related decline in mean arterial and venous plasma pH during submaximal exercise changed differently with the training intervention in SET_Saline_ compared with SET_NAC_ (time × leg interaction; arterial pH, *p* = 0.041; venous pH, *p* = 0.022) (Figure 5). While mean arterial plasma pH during submaximal exercise was non-significantly lower after the intervention in SET_Saline_ (*p* = 0.055), it did not change with training in SET_NAC_ (*p* = 0.627). Likewise, the mean venous plasma pH during submaximal exercise did not change with training in SET_Saline_ (*p* = 0.418), but it was higher after training in SET_NAC_ (*p* = 0.001). At exhaustion, a non-significant difference in the training-induced change in arterial and venous plasma pH was observed between SET_Saline_ and SET_NAC_ (time × leg interaction; arterial pH, *p* = 0.088; venous pH, *p* = 0.065). Specifically, arterial plasma pH at exhaustion was non-significantly lower after training in SET_Saline_ (−0.129 to 0.001; *p* = 0.053) but not in SET_NAC_ (*p* = 0.588), and the venous plasma pH at exhaustion was 0.058 lower after training in SET_Saline_ (−0.106 to −0.010; *p* = 0.022) but not in SET_NAC_ (*p* = 0.952). In recovery, mean arterial and venous plasma pH changed differently with the training intervention in SET_Saline_ and SET_NAC_ (time × leg interaction; arterial pH, *p* = 0.001; venous pH, *p* = 0.001). Specifically, the mean arterial plasma pH during recovery was lower after training in SET_Saline_ (*p* < 0.001) but not in SET_NAC_ (*p* = 0.228), and the mean venous plasma pH during recovery was lower after training in SET_Saline_ (*p* < 0.001) but not in SET_NAC_ (*p* = 0.408).

The mean venous-arterial plasma pH difference during submaximal exercise was non-significantly lower and lower after the training intervention in SET_Saline_ (*p* = 0.081) and SET_NAC_ (*p* < 0.001), respectively. No other training-induced changes were observed in the venous-arterial plasma pH difference in SET_Saline_ or SET_NAC_.

**Figure 6:**
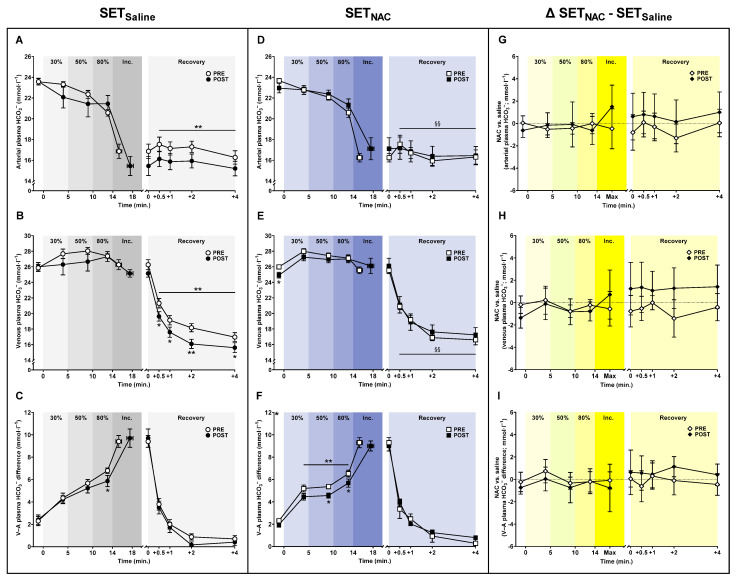
Effect of speed endurance training (SET) on plasma HCO_3_^−^ shifts without and with *N*-acetylcysteine infusion. Plasma HCO_3_^−^ concentrations (**A**–**F**) before, during, and after knee-extensor exercise without (SET_Saline_, n = 10) and with *N*-acetylcysteine (SET_NAC_, n = 9) infusion as well as differences (Δ) between SET_NAC_ and SET_Saline_ (**G**–**I**, n = 9) before (pre) and after (post) SET. ** Post different from pre (*p* < 0.01). * Post different from pre (*p* < 0.05). ^§§^ Trial × leg effect (*p* < 0.01). Data are presented as mean ± SEM (SET_Saline_ and SET_NAC_) or mean ± 95% CI (Δ SET_NAC_ − SET_Saline_).

The mean net leg H^+^ release during submaximal exercise was lower after the training intervention in both SET_Saline_ (*p* = 0.010) and SET_NAC_ (*p* = 0.003). Specifically, the net leg H^+^ exchange was 2.5 mmol·min^−1^ (−4.1 to −1.0; *p* = 0.005) lower at 80% W_max_ after the training intervention in SET_Saline_, and 1.6 mmol·min^−1^ (−3.0 to −0.2; *p* = 0.033) and 1.0 mmol·min^−1^ (−1.9 to 0.0; *p* = 0.045) lower at 50 and 80% W_max_, respectively, after the training intervention in SET_NAC_ (Figure 6D,H). However, in recovery, the mean net leg H^+^ exchange changed differently with training in SET_Saline_ compared to SET_NAC_ (time × leg interaction; *p* = 0.017), being non-significantly lower after training in SET_Saline_ (*p* = 0.055), and unaffected by training in SET_NAC_ (*p* = 0.101).

### 3.6. Plasma HCO_3_^−^ Shifts

The arterial plasma [HCO_3_^−^] during submaximal exercise was not affected by the training intervention in either SET_Saline_ or SET_NAC_, while the mean venous plasma [HCO_3_^−^] during submaximal exercise was non-significantly lower after training in SET_Saline_ (*p* = 0.091) and SET_NAC_ (*p* = 0.072) (Figure 6). In recovery, mean arterial and venous plasma [HCO_3_^−^] changed differently with the training intervention in SET_Saline_ and SET_NAC_ (time × leg interaction; arterial [HCO_3_^−^], *p* = 0.006; venous [HCO_3_^−^], *p* = 0.006). Specifically, mean arterial plasma [HCO_3_^−^] was lower after training in SET_Saline_ (all *p* < 0.001) but not in SET_NAC_ (*p* = 0.841), and mean venous plasma [HCO_3_^−^] was 1.7 (−3.0 to −0.4; *p* = 0.015), 1.5 (−2.7 to −0.4; *p* = 0.012), 2.1 (−3.1 to −1.1; *p* = 0.001), and 1.4 mmol·L^−1^ (−2.5 to −0.2; *p* = 0.022) lower (*p* < 0.001) at 0.5, 1, 2, and 4 min into recovery, respectively, after training in SET_Saline_, with no changes observed in SET_NAC_.

The mean venous-arterial plasma [HCO_3_^−^] difference during submaximal exercise was non-significantly lower and lower after training in SET_Saline_ (*p* = 0.072) and SET_NAC_ (*p* < 0.001), respectively. The venous-arterial plasma [HCO_3_^−^] difference was 0.9 mmol·L^−1^ (−1.7 to −0.1; *p* = 0.031) lower at 80% W_max_ after training in SET_Saline_, while it was 0.8 mmol·L^−1^ (−1.4 to −0.2; *p* = 0.014) and 0.8 mmol·L^−1^ (−1.6 to −0.1; *p* = 0.032) lower at 50 and 80% W_max_, respectively, after training in SET_NAC_.

### 3.7. Plasma K^+^ Shifts

**Figure 7:**
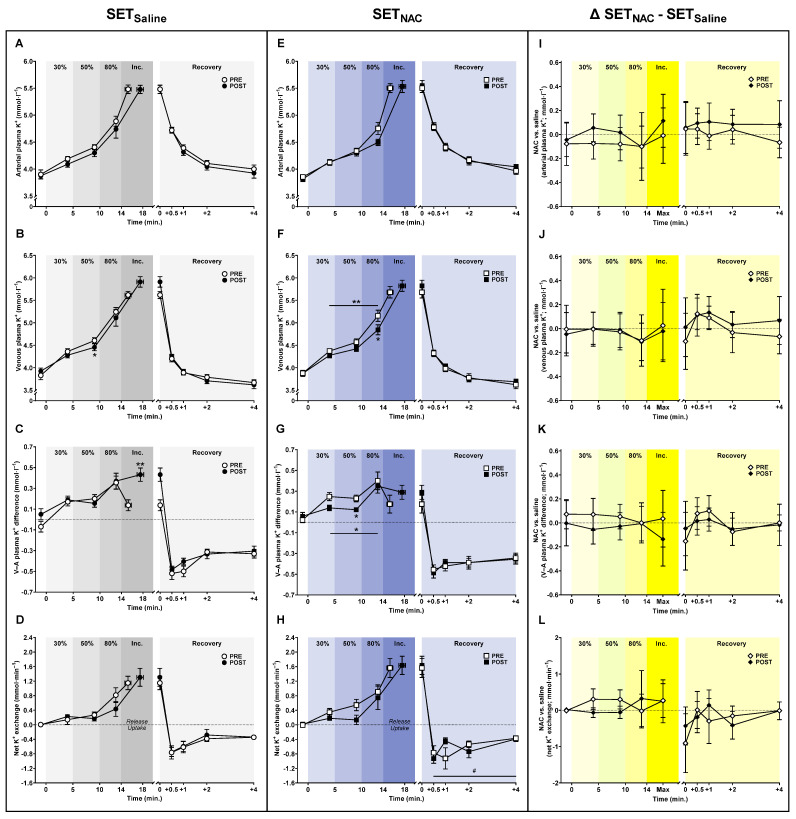
Effect of speed endurance training (SET) on plasma K^+^ shifts without and with *N*-acetylcysteine infusion. Plasma K^+^ concentrations (**A**–**C**,**E**–**G**) and net leg K^+^ exchange (**D**,**H**) before, during, and after knee-extensor exercise without (SET_Saline_, n = 10) and with *N*-acetylcysteine (SET_NAC_, n = 9) infusion as well as differences (Δ) between SET_NAC_ and SET_Saline_ (**I**–**L**, n = 9) before (pre) and after (post) SET. ** Post different from pre (*p* < 0.01). * Post different from pre (*p* < 0.05). ^#^ Trial × time effect (*p* < 0.05). Data are presented as mean ± SEM (SET_Saline_ and SET_NAC_) or mean ± 95% CI (Δ SET_NAC_ − SET_Saline_).

The mean arterial plasma [K^+^] during submaximal exercise was non-significantly lower after training in both SET_Saline_ (*p* = 0.052) and SET_NAC_ (*p* = 0.063) (Figure 7). Specifically, arterial plasma [K^+^] was non-significantly lower by 0.1 mmol·L^−1^ (−0.2 to 0.0; *p* = 0.086) and 0.1 mmol·L^−1^ (−0.2 to 0.0; *p* = 0.094) at 30 and 50% W_max_, respectively, after training in SET_Saline_, while it was non-significantly lower by 0.3 mmol·L^−1^ (−0.6 to 0.0; *p* = 0.076) at 80% W_max_ after training in SET_NAC_. The mean venous plasma [K^+^] during submaximal exercise was non-significantly lower and lower after training in SET_Saline_ (*p* = 0.091) and SET_NAC_ (*p* = 0.001), respectively. Specifically, venous plasma [K^+^] was 0.2 mmol·L^−1^ (−0.3 to 0.0; *p* = 0.011) lower at 50% W_max_ after training in SET_Saline_, and it was 0.3 mmol·L^−1^ (−0.6 to 0.0; *p* = 0.031) lower at 80% W_max_ after training in SET_NAC_. At exhaustion, venous plasma [K^+^] was non-significantly higher by 0.3 mmol·L^−1^ (0.0 to 0.6; *p* = 0.071) after training in SET_Saline_, with no training-induced changes in SET_NAC_ (*p* = 0.371). The rate of decline in venous plasma [K^+^] from exhaustion and 0.5 min into recovery increased by 0.3 mmol·L^−1^ (0.0 to 0.5; *p* = 0.040) with training in SET_Saline_, whereas it did not change in SET_NAC_ (*p* = 0.120). No changes were observed in venous plasma [K^+^] during recovery.

The mean venous-arterial plasma [K^+^] difference during submaximal exercise was lower after training in SET_NAC_ only (*p* = 0.024). The venous-arterial plasma [K^+^] difference was 0.1 mmol·L^−1^ (−0.2 to 0.0; *p* = 0.054) and 0.1 mmol·L^−1^ (−0.2 to 0.0; *p* = 0.013) lower at 30 and 50% W_max_, respectively, after training in SET_NAC_. However, at exhaustion, the venous-arterial plasma [K^+^] difference was 0.3 mmol·L^−1^ (0.1 to 0.5; *p* = 0.009) higher after training in SET_Saline_, but was unchanged in SET_NAC_ (*p* = 0.319). The rate of decline in the venous-arterial plasma [K^+^] difference from exhaustion and 0.5 min into recovery was 0.2 mmol·L^−1^ (0.0 to 0.4; *p* = 0.040) greater after training in SET_Saline_, with no training-induced change observed in SET_NAC_ (*p* = 0.537). No changes were observed in the venous-arterial plasma [K^+^] difference during recovery.

The mean net leg K^+^ release during submaximal exercise was unaffected by training in SET_Saline_ (*p* = 0.216), whereas it was non-significantly lower after training in SET_NAC_ (*p* = 0.076). However, in SET_NAC_, recovery net leg K^+^ exchange time points were different after training (trial × time effect; *p* = 0.038). No other changes were observed in net leg K^+^ exchange.

### 3.8. Plasma Na^+^ Shifts

**Figure 8:**
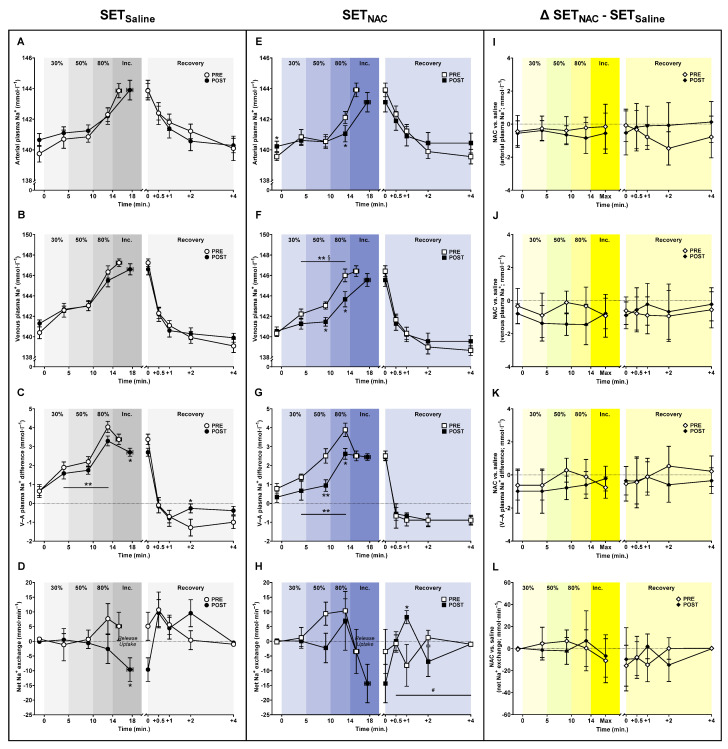
Effect of speed endurance training (SET) on plasma Na^+^ shifts without and with *N*-acetylcysteine infusion. Plasma Na^+^ concentrations (**A**–**C**,**E**–**G**) and net leg Na^+^ exchange (**D**,**H**) before, during, and after knee-extensor exercise without (SET_Saline_, n = 10) and with *N*-acetylcysteine (SET_NAC_, n = 9) infusion as well as differences (Δ) between SET_NAC_ and SET_Saline_ (**I**–**L**, n = 9) before (pre) and after (post) SET. ** Post different from pre (*p* < 0.01). * Post different from pre (*p* < 0.05). ^#^ Trial × time effect (*p* < 0.05). ^§^ Trial × leg effect (*p* < 0.05). Data are presented as mean ± SEM (SET_Saline_ and SET_NAC_) or mean ± 95% CI (Δ SET_NAC_ − SET_Saline_).

The mean arterial plasma [Na^+^] during submaximal exercise did not change with training in either SET_Saline_ (*p* = 0.529) or SET_NAC_ (*p* = 0.104). However, arterial plasma [Na^+^] was 1.1 mmol·L^−1^ (−2.1 to 0.0; *p* = 0.047) lower at 80% W_max_ after training in SET_NAC_ (Figure 8). No other changes were observed in arterial plasma [Na^+^]. The exercise-related increase in mean venous plasma [Na^+^] during submaximal exercise changed differently with the training intervention in SET_Saline_ and SET_NAC_ (time × leg interaction; *p* = 0.009), with mean venous plasma [Na^+^] being unaffected by training in SET_Saline_ (*p* = 0.474), but being 1.6 mmol·L^−1^ (−2.8 to −0.4; *p* = 0.013) and 2.3 mmol·L^−1^ (−4.0 to −0.6; *p* = 0.013) lower at 50 and 80% W_max_, respectively, after training in SET_NAC_.

The mean venous-arterial plasma [Na^+^] difference during submaximal exercise was lower after training in both SET_Saline_ (*p* = 0.009) and SET_NAC_ (*p* < 0.001). Specifically, the venous-arterial plasma [Na^+^] difference at 80% W_max_ was non-significantly lower by 0.7 mmol·L^−1^ (−1.5 to 0.0; *p* = 0.056) after training in SET_Saline_, whereas it was 1.6 mmol·L^−1^ (−2.5 to −0.6; *p* = 0.004) and 1.3 mmol·L^−1^ (−2.2 to −0.3; *p* = 0.0015) lower at 50 and 80% W_max_, respectively, after training in SET_NAC_. At exhaustion, the venous-arterial plasma [Na^+^] difference was 0.7 mmol·L^−1^ (−1.1 to −0.2; *p* = 0.008) lower after training in SET_Saline_, while it was 1 mmol·L^−1^ (0.0 to 2.0; *p* = 0.049) higher 2 min into recovery.

The mean net leg Na^+^ exchange during submaximal exercise did not change with the training intervention in either SET_Saline_ (*p* = 0.300) or SET_NAC_ (*p* = 0.234). At exhaustion, the mean net leg Na^+^ exchange was 14.7 mmol·min^−1^ (−27.7 to −1.8; *p* = 0.029) lower after training in SET_Saline_, with no training-induced change in SET_NAC_ (*p* = 0.235). The mean net leg Na^+^ exchange was 16.4 mmol·min^−1^ (0.4 to 32.3; *p* = 0.046) higher at 2 min into recovery after training in SET_NAC_, with the time points in recovery being different after training (trial × time effect; *p* = 0.019).

### 3.9. Cumulated Leg Lactate^−^, H^+^, K^+^, and Na^+^ Exchange during Submaximal and Intense Exercise

The cumulated net leg lactate^−^ release during submaximal exercise was 6.8 mmol (−10.3 to −3.3; *p* = 0.001) and 8.1 mmol (−13.2 to −3.0; *p* = 0.006) lower after training in SET_Saline_ and SET_NAC_, respectively (Table 3). The cumulated net leg lactate^−^ release during the incremental part of the exercise to exhaustion was 7.7 mmol (4.0 to 11.4; *p* = 0.001) and 8.6 mmol (4.7 to 12.5; *p* = 0.001) higher after training in SET_Saline_ and SET_NAC_, respectively.

The cumulated net leg H^+^ release during submaximal exercise was 7.9 mmol (−15.5 to −0.3; *p* = 0.044) and 9.3 mmol (−17.6 to −1.0; *p* = 0.032) lower after training in SET_Saline_ and SET_NAC_, respectively. The cumulated net leg H^+^ release during the incremental part of the exercise to exhaustion was 7.5 mmol (5.5 to 9.5; *p* < 0.001) and 11.4 mmol (4.1 to 18.7; *p* = 0.006) higher after training in SET_Saline_ and SET_NAC_, respectively.

The cumulated net leg K^+^ release and Na^+^ exchange during exercise were not affected by training either in SET_Saline_ or SET_NAC_.

### 3.10. Muscle Lactate^−^ and pH at Exhaustion and Buffer Capacity

**Figure 9:**
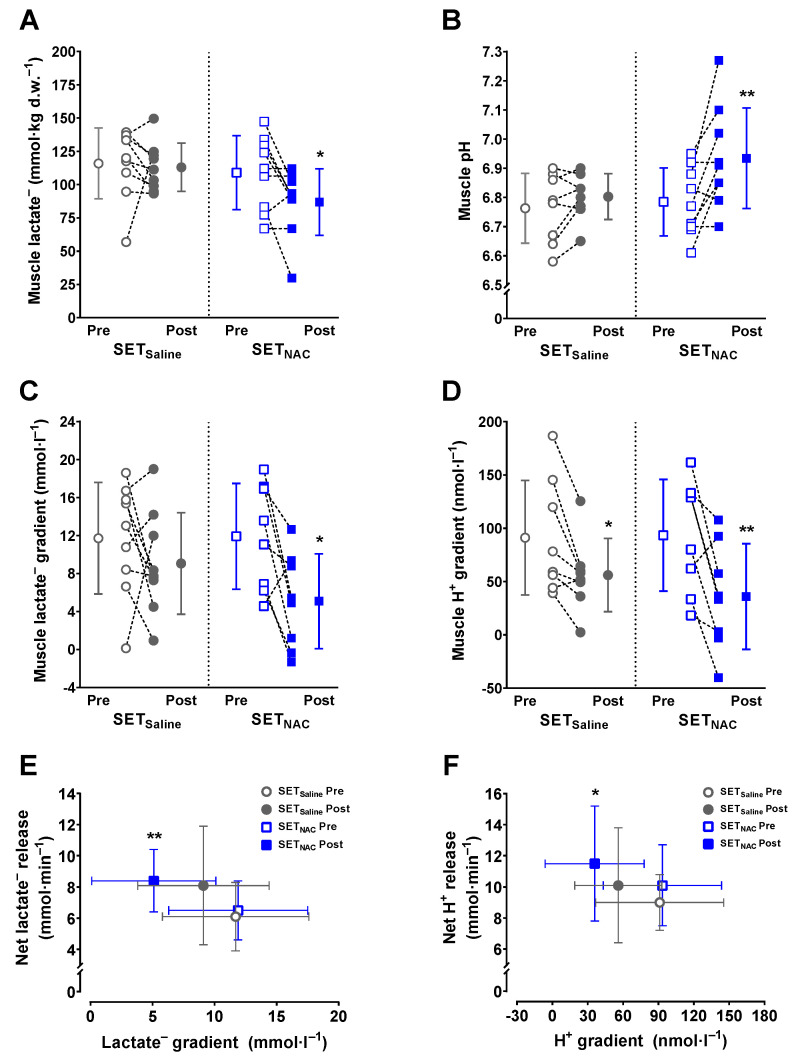
Effect of speed endurance training (SET) on muscle lactate^−^ and pH at exhaustion without and with *N*-acetylcysteine infusion. Muscle [lactate^−^] ((**A**), SET_Saline_, n = 9; SET_NAC_, n = 8), muscle pH ((**B**), SET_Saline_, n = 8; SET_NAC_, n = 8), muscle [lactate^−^] gradient ((**C**), SET_Saline_, n = 9; SET_NAC_, n = 8), muscle [H^+^] gradient ((**D**), SET_Saline_, n = 8; SET_NAC_, n = 8) and the relationship between muscle gradients and net release ((**E**,**F**)*,* SET_Saline_, n = 9; SET_NAC_, n = 8) immediately after knee-extensor exercise to exhaustion without (SET_Saline_) and with *N*-acetylcysteine (SET_NAC_) infusion before (pre) and after (post) SET. ** Post different from pre (*p* < 0.01). * Post different from pre (*p* < 0.05). Data are presented as mean ± SD with individual changes (**A**–**D**) or as mean ± SD (**E**,**F**).

Muscle [lactate^−^] and muscle pH at exhaustion were not affected by training in SET_Saline_, whereas they were 25 mmol·kg d.w.^−1^ (−43 to −7; *p* = 0.012) lower and 0.19 mmol·kg d.w.^−1^ (0.08 to 0.29; *p* = 0.003) higher, respectively, in SET_NAC_ (Figure 9A,B). The muscle [lactate^−^] gradient at exhaustion was 6.8 mmol·L^−1^ (−11.6 to −2.1; *p* = 0.010) lower after training in SET_NAC_, but not in SET_Saline_ (*p* = 0.295) (Figure 9C). The muscle [H^+^] gradient was 35 nmol·L^−1^ (−58 to −12; *p* = 0.008) and 57 nmol·L^−1^ (−94 to −21; *p* = 0.007) lower after training in SET_Saline_ and SET_NAC_, respectively (Figure 9D). The relationship between the muscle [lactate^−^] gradient and peak net leg lactate^−^ release (*p* = 0.115) as well as the muscle [H^+^] gradient and peak net leg H^+^ release (*p* = 0.098) were not affected by training in SET_Saline_. However, in SET_NAC_, the training intervention changed the relationship between the muscle [lactate^−^] gradient and peak net leg lactate^−^ release (*p* = 0.003), as well as the muscle [H^+^] gradient and peak net leg H^+^ release (*p* = 0.020), so that a lower muscle concentration gradient resulted in a greater peak net leg release (Figure 9E,F). No change was observed in muscle buffer capacity, being 143 ± 14 and 154 ± 36 mmol H^+^·kg d.w.^−1^·pH^−1^ in SET_Saline_ (*p* = 0.207, n = 7) and 146 ± 13 and 142 ± 12 mmol H^+^·kg d.w.^−1^·pH^−1^ in SET_NAC_ (*p* = 0.595, n = 7) before and after the training intervention, respectively.

### 3.11. Muscle Content of Ion-Handling Proteins and Antioxidant Enzymes

**Figure 10:**
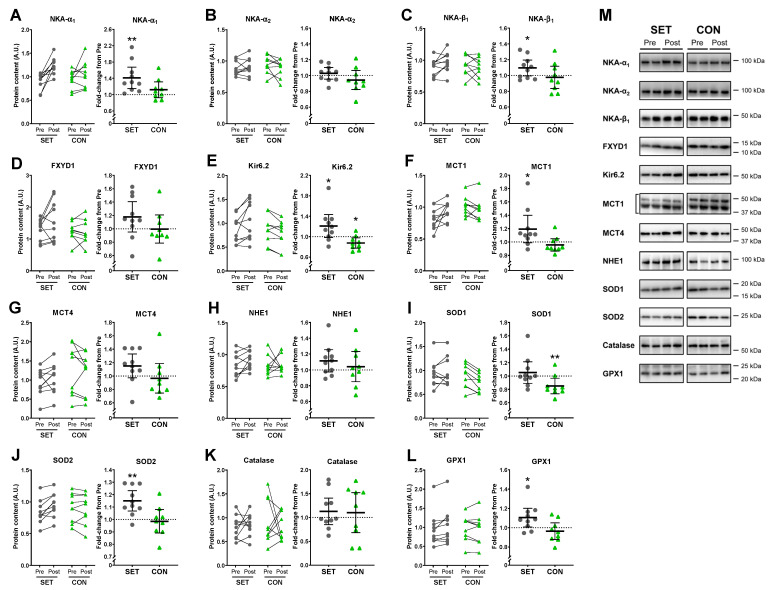
Effect of speed endurance training (SET) on the muscle content of ion-handling proteins and antioxidant enzymes. Change in the muscle content of ion-handling proteins (**A**–**H**) and antioxidant enzymes (**I**–**L**) as well as representative blots (**M**) before (pre) to after (post) the SET intervention (SET, n = 10) or habitual lifestyle maintenance (CON, n = 9). NKA, Na^+^/K^+^-ATPase; FXYD1, regulatory subunit of NKA; Kir6.2, ATP-sensitive K^+^-channel subunit Kir6.2; MCT, monocarboxylate transporter; NHE1, Na^+^/H^+^ exchanger; SOD, superoxide dismutase; GPX, glutathione peroxidase. ** Post different from pre (*p* < 0.01). * Post different from pre (*p* < 0.01). Data are presented as individual pre and post values and as mean fold change ± 95% CI relative to pre with individual changes.

At baseline (Pre), the muscle content of ion-handling proteins and antioxidant enzymes was similar in SET and CON. In SET, the training intervention increased the muscle content of NKA-α_1_ by 41% (15 to 67; *p* = 0.001), NKA-β_1_ by 10% (0 to 20; *p* = 0.050), Kir6.2 by 21% (−2 to 43; *p* = 0.042), MCT1 by 19% (−1 to 40; *p* = 0.028), SOD2 by 15% (7 to 23; *p* = 0.001), and GPX1 by 11% (1 to 20; *p* = 0.023) (Figure 10). In addition, the muscle content of NHE1 increased non-significantly by 11% (−3 to 25; *p* = 0.087) after SET. In CON, muscle content of Kir6.2 decreased by 12% (−22 to −2; *p* = 0.014) and SOD1 by 15% (−27 to −3; *p* = 0.004). No other changes were observed in SET or CON.

### 3.12. Muscle Phosphorylation of FXYD1

**Figure 11:**
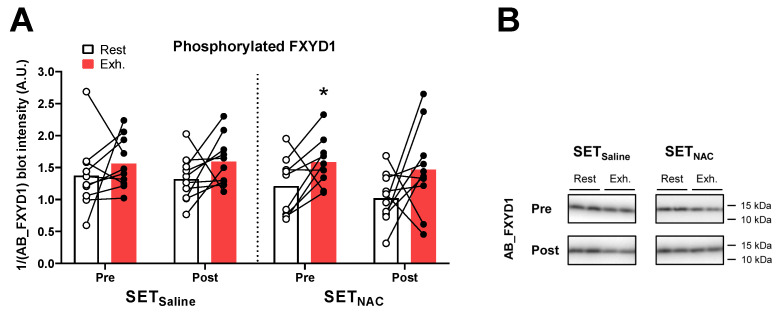
Effect of speed endurance training (SET) on the muscle content of ion-handling proteins and antioxidant enzymes. Phosphorylated FXYD1 (**A**) and representative blots (**B**) at rest and after knee-extensor exercise to exhaustion (exh.) without (SET_Saline_, n = 10) and with *N*-acetylcysteine (SET_NAC_, pre n = 9, post n = 10) infusion before (pre) and after (post) SET. * Exh. different from rest (*p* < 0.05). Data are presented as mean with individual changes.

Before the training intervention, phosphorylation of FXYD1 increased non-significantly from rest to exhaustion by 14% (−7 to 14; *p* = 0.226) in SET_Saline_, whereas it increased significantly by 31% (6 to 31; *p* = 0.034) in SET_NAC_ (Figure 11). After the training intervention, phosphorylation of FXYD1 increased non-significantly from rest to exhaustion by 21% (0 to 21; *p* = 0.104) and 44% (−7 to 44; *p* = 0.347) in SET_Saline_ and SET_NAC_, respectively.

### 3.13. Mitochondrial Respiratory Capacity

**Figure 12:**
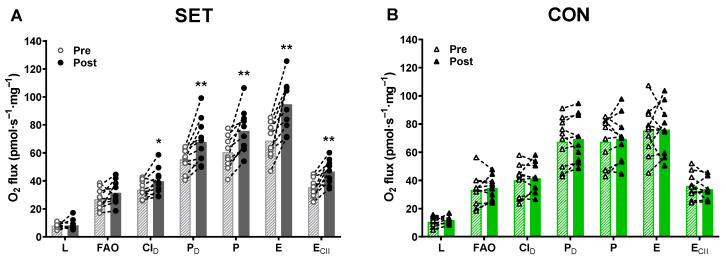
Effect of speed endurance training (SET) on mitochondrial respiratory capacity. Mitochondrial respiratory capacity at different states before (pre) and after (post) SET ((**A**), SET, n = 10) or habitual lifestyle maintenance ((**B**), CON, n = 10). L, leak respiration; FAO, fatty acid oxidation; CI_D_, complex I-linked respiration at sub-saturating ADP concentration; P_D_, complex I+II linked respiration at sub-saturating ADP concentration; P, maximal complex I+II linked respiration; E, maximal electron transfer-pathway capacity; E_CII_, complex II-linked electron transfer-pathway capacity. ** Post different from pre (*p* < 0.01). * Post different from pre (*p* < 0.05). Data are presented as mean with individual changes.

In SET, the training intervention increased FAO non-significantly by 17% (−5 to 39; *p* = 0.076), whereas mitochondrial respiratory capacity increased during CI_D_ by 18% (−2 to 38; *p* = 0.020), P_D_ by 22% (2 to 43; *p* = 0.004), *p* by 25% (7 to 43; *p* = 0.001), E by 38% (19 to 57; *p* < 0.001), and E_CII_ by 31% (14 to 48; *p* = 0.001) (Figure 12). No changes were observed in CON.

### 3.14. Relationship between Training-Induced Changes in Main Outcomes

No significant correlations were observed between change percentages induced by SET in main outcomes and selected explanatory variables (Table 4).

## 4. Discussion

The major finding of this study was that a period of SET altered the effect of *N*-acetylcysteine on muscle ionic shifts during exercise in young males. Specifically, after but not before SET, *N*-acetylcysteine lowered shifts of plasma H^+^, HCO_3_^−^, and Na^+^ during submaximal exercise and in recovery from intense exercise, while also enhancing the ability to extrude lactate^−^ and H^+^ against a lower concentration gradient across the exercising leg. Despite these *N*-acetylcysteine-dependent alterations in exercise-related ionic shifts after SET, improvements in intense exercise performance elicited by SET were not magnified by *N*-acetylcysteine (SET_NAC_ vs. SET_Saline_). The present study also demonstrates that SET effectively enhances the ion transport capacity of skeletal muscle. This is substantiated by lowered exercise-related shifts of plasma lactate^−^, H^+^, K^+^, and Na^+^ during submaximal exercise after SET, and increased peak net leg lactate^−^ release and Na^+^ uptake. These changes were associated with a greater abundance of ion-handling proteins (NKA-α_1_, NKA-β_1_, Kir6.2, and MCT1) and antioxidant enzymes (SOD2 and GPX1) as well as enhanced mitochondrial respiratory capacity and increased lean mass of the trained muscle after SET.

A key finding of the present study was that *N*-acetylcysteine infusion only affected exercise-related ionic shifts after and not before SET, as reflected by lower shifts of plasma pH, Na^+^_,_ and HCO_3_^−^ during submaximal exercise and in recovery from intense exercise, but also possessed a superior ability to extrude lactate^−^ and H^+^ against a lower concentration gradient when approaching exhaustion from intense exercise. This observation confirms presumptions from between-study comparisons of *N*-acetylcysteine being training-dependent [16,17,18]. Our observation of *N*-acetylcysteine eliciting greater effects after SET despite a supposedly increase in intrinsic ROS-scavenging capacity, as indicated by the upregulation of SOD2 and GPX1 muscle content, points to a SET-induced increase of reduced glutathione in skeletal muscle. This assumption is supported by animal models displaying greater reduced glutathione levels in skeletal muscle following exercise training [39,40] and by human data indicating that trained individuals exhibit greater muscle content of reduced glutathione after *N*-acetylcysteine infusion [18].

In line with previous studies [7,10], we observed that SET increased the capacity for muscle lactate^−^ and H^+^ release during intense exercise, ultimately resulting in similar levels of muscle lactate^−^ and H^+^ at exhaustion despite the prolonged exercising time after SET. Because we observed no change in muscle NHE1 abundance, this suggests that the training-induced changes in muscle lactate^−^ and H^+^ handling during intense exercise were independent of NHE1-related mechanisms. The lowered lactate^−^ and H^+^ shifts during submaximal exercise after SET’ however, this likely not only reflects the greater abundance of MCT1, but also the enhanced mitochondrial respiratory capacity and the increased mass of the trained muscle. Such adaptations lower the reliance on anaerobic energetic pathways by respectively pushing the capacity for aerobic energy production and lowering the relative intensity of the submaximal exercise [4].

Despite the longer time to exhaustion after SET, the peak net leg K^+^ release was similar before and after SET, which suggests that SET enhanced K^+^ reuptake by the exercising muscle. This may involve several adaptive mechanisms. Firstly, SET increased the muscle content of NKA-α_1_ and NKA-β_1_, thereby enlarging the pool for NKA pump assembly and sarcolemmal translocation [41]. Secondly, the activation of FXYD1 measured immediately after exercise was more pronounced after SET, likely resulting in enhanced NKA activity and, thus, increased K^+^ reuptake [42]. Thirdly, the SET-induced increase in SOD2 and GPX1 abundance possibly enhanced ROS-scavenging capacity, ultimately reducing ROS-dependent inhibitory effects on NKA activity. Lastly, the greater Na^+^ uptake by the exercising muscle observed after SET may be associated with an increased NKA activity, which would ultimately promote myoplasmic K^+^ reuptake.

The above mechanisms, along with the greater capacity for aerobic energy production, possibly explain the lower venous plasma [K^+^] observed during submaximal exercise after SET. Indeed, a lower relative exercise intensity after SET would reduce motor unit firing frequency and the amount of K^+^ released from the contracting fibres. Furthermore, the ability to counter K^+^ shifts during submaximal exercise may be related to improved metabolic stability, as indicated by lowered lactate^−^ and H^+^ shifts, since metabolic perturbations such as lowered muscle adenosine nucleotide content and pH can exacerbate K^+^ efflux by increasing Kir6.2 opening probability [43]. Collectively, the observed SET-induced reduction in lactate^−^ and H^+^ shifts during submaximal exercise may be due to improved mitochondrial respiratory capacity and lowered relative exercise intensity, also contributing to lower K^+^ release.

As expected, SET markedly improved intense exercise performance, as measured by time to exhaustion during incremental knee-extensor exercise [3]. However, the SET-induced enhancement of performance was similar for the leg receiving saline as for the leg receiving *N*-acetylcysteine infusion. This occurred although the accumulation of muscle [lactate^−^] and [H^+^] at exhaustion was of a lower magnitude after SET only in the leg receiving *N*-acetylcysteine infusion, suggesting that accumulation of myocellular lactate^−^ and H^+^ per se does not cause fatigue during intense exercise, as also previously suggested [44]. Conversely, the observed SET-induced increase in K^+^ reuptake capacity was likely of major importance, as excessive extracellular K^+^ accumulation can cause sarcolemma inexcitability, thereby contributing to muscle fatigue [1,2,3,4,5].

## 5. Conclusions

This study demonstrates that a period of SET potentiates the effect *N*-acetylcysteine with respect to its action on exercise-related ionic shifts in young males. This supports a training dependency for *N*-acetylcysteine responsiveness. In addition, the present results indicate that SET enhances the ability to counter ionic shifts during exercise via a multitude of factors not only related to upregulation of muscle ion handling proteins but also antioxidant enzymes and greater mitochondrial respiratory capacity of the trained muscle.

## Figures and Tables

**Table 1:** Subject characteristics at inclusion.

	SET (n = 10)	CON (n = 10)
Age (yr)	23.0 ± 3.4	25.7 ± 3.7
Height (cm)	186 ± 6	184 ± 6
Body mass (kg)	79.6 ± 12.1	76.0 ± 8.1
BMI (kg·m^−2^)	23.0 ± 2.5	22.4 ± 1.6
V̇O_2max_ (mL·min^−1^·kg^−1^)	50.2 ± 4.5	52.8 ± 6.4

SET, speed endurance training group; CON, control group; BMI, body mass index; V̇O_2max_, maximal oxygen consumption. Data are presented as mean ± SD.

**Table 2:** Whole-body and thigh composition before and after speed endurance training (SET) or habitual lifestyle maintenance (CON).

Whole-Body		SET		CON	
Body mass (kg)	Pre	79.6 ± 12.1		76.0 ± 8.1	
	Post	79.5 ± 11.6		75.9 ± 8.1	
Body fat mass (kg) ^#^	Pre	16.2 ± 5.3		11.6 ± 3.4	
	Post	15.6 ± 4.9		11.5 ± 3.6	
Body fat percent (%)	Pre	20.0 ± 4.8		15.4 ± 5.2	
	Post	19.4 ± 4.7 *		15.2 ± 5.2	
Body lean mass (kg)	Pre	60.4 ± 9.0		61.6 ± 8.6	
	Post	60.9 ± 8.9		61.5 ± 8.4	
**Thigh**		**SET_Saline_**	**SET_NAC_**	**CON_Saline_**	**CON_NAC_**
Thigh mass (g)	Pre	8817 ± 1621	8929 ± 1478	8532 ± 1220	8299 ± 1104
	Post	8955 ± 1549	9085 ± 1380 *	8497 ± 1244	8256 ± 1056
Thigh fat mass (g)	Pre	1609 ± 543	1650 ± 536	1209 ± 380	1190 ± 372
	Post	1566 ± 526	1606 ± 524	1198 ± 419	1194 ± 409
Thigh fat percent (%)	Pre	18.1 ± 4.2	18.4 ± 4.6	14.3 ± 4.8	14.6 ± 5.1
	Post	17.3 ± 4.3 *	17.6 ± 4.6	14.2 ± 4.9	14.6 ± 5.1
Thigh lean mass (g)	Pre	6943 ± 1263	7013 ± 1196	7054 ± 1158	6846 ± 1104
	Post	7125 ± 1242 **	7214 ± 1136 **	7032 ± 1138	6799 ± 1019

SET (n = 10), speed endurance training group; CON (n = 10), control group; SET_Saline_ (, n = 10), right leg in SET; SET_NAC_ (n = 10), left leg in SET; CON_Saline_ (n = 10), right leg in CON; CON_NAC_ (n = 10), left leg in CON. ^#^ CON different from SET (*p* < 0.05). ** Post different from Pre (*p* < 0.01). * Post different from Pre (*p* < 0.05). Data are presented as mean ± SD; n = 10.

**Table 3:** Effect of speed endurance training (SET) on cumulated net leg lactate^−^, H^+^, K^+^, and Na^+^ exchange during submaximal and intense exercise.

		Lactate^−^		H^+^		K^+^		Na^+^	
		SET_Saline_	SET_NAC_	SET_Saline_	SET_NAC_	SET_Saline_	SET_NAC_	SET_Saline_	SET_NAC_
Submaximal exercise (mmol)	Pre	21.9 ± 8.3	23.0 ± 9.8	50.3 ± 15.6	57.1 ± 12.1	3.2 ± 2.2	4.9 ± 2.3	12.5 ± 53.7	41.6 ± 43.0
	Post	15.1 ± 5.7 **	14.9 ± 8.2**	42.4 ± 16.9 *	47.8 ± 10.2 *	2.1 ± 2.3	2.5 ± 3.3	−5.8 ± 45.7	−2.6 ± 119.0
Submaximal exercise (mmol·min^−1^)	Pre	1.6 ± 0.6	1.7 ± 0.7	3.7 ± 1.0	4.1 ± 0.9	0.2 ± 0.2	0.3 ± 0.2	1.0 ± 4.1	3.0 ± 3.1
	Post	1.1 ± 0.4 **	1.1 ± 0.6 **	3.1 ± 1.2 *	3.4 ± 0.7 *	0.2 ± 0.2	0.2 ± 0.2	−0.4 ± 3.3	−0.1 ± 8.6
Incremental exercise to exh. (mmol)	Pre	4.2 ± 2.8	5.4 ± 4.6	6.7 ± 4.6	7.9 ± 5.5	0.6 ± 0.4	0.9 ± 0.7	−6.3 ± 38.3	−2.1 ± 8.4
	Post	11.8 ± 5.6 **	14.0 ± 6.1 **	14.2 ± 5.0 **	19.4 ± 7.0 **	1.1 ± 1.7	1.2 ± 2.6	−17.0 ± 62.7	−21.4 ± 99.3
Incremental exercise to exh. (mmol·min^−1^)	Pre	2.2 ± 1.3	2.6 ± 1.4	3.4 ± 1.9	3.6 ± 1.6	0.4 ± 0.3	0.4 ± 0.3	0.6 ± 15.1	−1.2 ± 5.2
	Post	3.0 ± 1.6	3.3 ± 1.6	3.6 ± 1.1	4.5 ± 1.4	0.3 ± 0.4	0.2 ± 0.7	−4.1 ± 14.3	−7.8 ± 29.3

Total net leg lactate^−^, H^+^, K^+^, and Na^+^ exchange during submaximal and intense exercise without (SET_Saline_, n = 10) and with *N*-acetylcysteine (SET_NAC_, n = 9) infusion before (Pre) and after (Post) six weeks of SET. Submaximal exercise consisted of 5 min at 30 and 50% W_max_, respectively, followed by 4 min at 80% W_max_. Intense exercise was the subsequent exercise duration after 80% W_max_, which consisted of increments in resistance of 6 W·min^−1^ until exhaustion. W_max_, leg peak power output; exh., exhaustion. ** Post different from pre (*p* < 0.01). * Post different from pre (*p* < 0.05). Data are presented as mean ± SD.

**Table 4:** Correlation for change percentages in SET_saline_ for main outcomes.

		Pearson’s Correlation Coefficient (r)	*p*-Value
Time to exhaustion	Muscle pH	−0.52	0.189
	Muscle lactate	0.00	0.994
	Leg lean mass	−0.61	0.062
	NKA-α_1_	0.48	0.161
	NKA-α_2_	0.53	0.139
	NKA-β_1_	−0.31	0.379
	FXYD1	0.04	0.914
Venous plasma K^+^ during submaximal exercise (measured at 80% W_max_)	Leg lean mass	−0.57	0.088
	NKA-α_1_	0.23	0.520
	NKA-α_2_	0.27	0.483
	NKA-β_1_	0.15	0.679
	FXYD1	0.01	0.985
Mean net leg K^+^ release during submaximal exercise (measured at 80% W_max_)	Leg lean mass	−0.56	0.092
	NKA-α_1_	0.17	0.640
	NKA-α_2_	0.32	0.399
	NKA-β_1_	−0.44	0.199
	FXYD1	0.17	0.632
Peak net leg K^+^ release at exhaustion	Leg lean mass	0.20	0.579

NKA, Na^+^, K^+^ ATPase; FXYD1, regulatory subunit of NKA.

## Data Availability

All data is contained within the article and supplementary materials.

## Supplementary Materials

The following supporting information can be downloaded at: https://www.mdpi.com/article/10.3390/antiox12010053/s1, Table S1. Primary antibodies used for western blotting.
