# Supplementary material for: High-Intensity Exercise Training Alters the Effect of N-Acetylcysteine on Exercise-Related Muscle Ionic Shifts in Men
Source: Antioxidants (Basel). 2022 Dec 27;12(1):53. doi: 10.3390/antiox12010053 (PMC9855150; doi:10.3390/antiox12010053)
Supplement: Supplementary file 1 [file antioxidants-12-00053-s001.zip › antioxidants-1914162-supplementary.pdf]

High-Intensity Exercise Training Alters the Effect of *N*-Acetylcysteine on Exercise-Related Muscle Ionic Shifts in Men

Anders K. Lemminger †, Matteo Fiorenza †, Kasper Eibye, Jens Bangsbo and Morten Hostrup *

The August Krogh Section for Human Physiology, Department of Nutrition, Exercise and Sports, University of Copenhagen, 1165 Copenhagen, Denmark
* Correspondence: mhostrup@nexs.ku.dk
† These authors contributed equally to this work.

Table S1. Primary antibodies used for western blotting.

Protein Target	Ab. cat. Number or Name	Company or Donor	Antibody Solution	Protein Loading Amount	Gel Type	Dilution
NKA-α_1	α6F	DSHB	3% BSA	9 µg	4-15%	1:500
NKA-α_2	07-674	Millipore	3% BSA	6 µg	4-15%	1:500
NKA-β_1	MA3-930	Thermo Fischer Scientific	2% milk	6 µg	4-15%	1:1000
FXYD1	13721-1AP	Proteintech	2% milk	9 µg	4-15%	1:2500
Kir6.2	SC-11228	Santa Cruz Biotechnology	2% milk	12 µg	4-15%	1:500
MCT1	SC-14916	Santa Cruz Biotechnology	3% BSA	12 µg	4-15%	1:250
MCT4	AB3316P	Sigma-Aldrich	2% milk	12 µg	4-15%	1:1000
NHE1	MAB3140	Millipore	3% BSA	12 µg	4-15%	1:500
SOD1	574597	Sigma-Aldrich	3% BSA	12 µg	4-15%	1:1000
SOD2	06-984	Sigma-Aldrich	2% milk	12 µg	4-15%	1:1000
Catalase	AB1877	Abcam	3% BSA	12 µg	4-15%	1:5000
GPX1	AB22604	Abcam	3% BSA	12 µg	4-15%	1:1000
FXYD1 unphosphorylated	AB_FXYD1–C2	Dr. J. Randall Moorman, University of Virginia	2% milk	12 µg	4-15%	1:5000

NKA, Na^+/K^+-ATPase; FXYD1, regulatory subunit of NKA; Kir6.2, ATP-sensitive K^+-channel subunit Kir6.2; MCT, monocarboxylate transporter; NHE1, Na^+/H^+ exchanger; SOD, superoxide dismutase; GPX, glutathione peroxidase; BSA, bovine serum albumin.
